# Mortality in relation to smoke exposure 9 years after the Hazelwood coal mine fire

**DOI:** 10.1093/ije/dyag071

**Published:** 2026-05-12

**Authors:** Karen Walker-Bone, Caroline X Gao, Catherine L Smith, Tingting Ye, David Brown, Natasha Kinsman, Jillian F Ikin, Matthew Carroll, Michael J Abramson, Yuming Guo, Tyler J Lane

**Affiliations:** School of Public Health and Preventive Medicine, Monash University, Melbourne, VIC, Australia; School of Public Health and Preventive Medicine, Monash University, Melbourne, VIC, Australia; Orygen, Centre for Youth Mental Health, The University of Melbourne, Parkville, VIC, Australia; School of Public Health and Preventive Medicine, Monash University, Melbourne, VIC, Australia; Orygen, Centre for Youth Mental Health, The University of Melbourne, Parkville, VIC, Australia; School of Public Health and Preventive Medicine, Monash University, Melbourne, VIC, Australia; School of Public Health and Preventive Medicine, Monash University, Melbourne, VIC, Australia; School of Public Health and Preventive Medicine, Monash University, Melbourne, VIC, Australia; School of Public Health and Preventive Medicine, Monash University, Melbourne, VIC, Australia; Monash Rural Health Churchill, Monash University, Churchill, VIC, Australia; School of Public Health and Preventive Medicine, Monash University, Melbourne, VIC, Australia; School of Public Health and Preventive Medicine, Monash University, Melbourne, VIC, Australia; School of Public Health and Preventive Medicine, Monash University, Melbourne, VIC, Australia

**Keywords:** all-cause mortality, cardiac mortality, coal mine fire, air pollution, particulate matter, PM_2.5_

## Abstract

**Background:**

In 2014, a coal mine fire in rural Australia shrouded nearby towns in smoke for several weeks. In response to community concerns and a lack of available evidence, the Victorian Department of Health commissioned a long-term health study. This article evaluates smoke effects on mortality 9 years after the fire.

**Methods:**

In 2016–17, 2872 individuals living near the mine during the fire were recruited into a cohort study and consented to linkage of their data. Time-location diaries were blended with estimates of hourly fire-related fine particulate matter ≤2.5 µm (PM_2.5_) to generate individual exposure measures. Mortality data were obtained up to mid-2023 by linkage with the National Death Index. Cardiac, cancer, and respiratory-related deaths were identifiable up to December 2021 and predicted thereafter to mid-2023. All-cause mortality effects were evaluated with Cox proportional-hazards models and cause-specific mortality with a competing risk survival model.

**Results:**

Nine years post-fire, 318 (11%) of the sample had died. All-cause mortality was not associated with PM_2.5_ exposure. There was no detectable effect on cancer deaths. However, a 10 µg/m^3^ increase in daily mean exposure to fire-related PM_2.5_ was associated with an 18% (95% CI: 2%–37%) higher risk of cardiac mortality, while PM_2.5_ exacerbated risk of respiratory mortality from tobacco use.

**Conclusion:**

Fire-related PM_2.5_ increased cardiac mortality and smoking effects on respiratory mortality years after the event. These findings concord with a growing body of evidence on the deadly risks of wildfire smoke. Protecting communities and individuals from smoke during and after large fire events should be a public health priority.

Key Messages

- We examined whether smoke exposure from a mine fire affected long-term mortality.
- Smoke increased cardiovascular mortality and exacerbated the effects of smoking on respiratory mortality, but no effects were observed on all-cause or cancer mortality.
- Exposure to smoke from extreme fire events may have long-term effects on mortality, which is a growing problem due to climate change.

## Introduction

During severe fire weather in mid-summer 2014, embers from a grass fire in regional Victoria, Australia, ignited the open-cut brown coal mine adjacent to the Hazelwood power station. The resultant coal mine fire burned for 6 weeks, enveloping nearby communities in dense smoke and ash. More than 7000 firefighters were deployed, 500 or so at a time, to suppress the fire and many were treated for smoke inhalation. Nearby residents reported suffering from numerous symptoms including headaches, blurred vision, cough, shortness of breath, epistaxis, fatigue, gastrointestinal symptoms, and chest pain. To investigate the long-term health effects of the fire, the Victorian Department of Health issued a request for tender, resulting in the establishment of the Hazelwood Health Study later that year [1].

Smoke from the mine fire contained levels of fine particulate matter ≤2.5 µm (PM_2.5_), carbon monoxide, and benzene that far exceeded national air quality standards, with PM_2.5_ concentrations the most persistently elevated [2, 3]. Estimated hourly PM_2.5_ concentrations [3] reached 3730 μg/m^3^, exceeding the Environment Protection Authority Victoria threshold for extremely poor air quality of 300 μg/m^3^ by more than 12 times [4]. There is considerable evidence that PM_2.5_ causes numerous health problems [5] including an increased risk of mortality [6, 7], and that PM_2.5_ is more deadly when it originates from landscape fires [8, 9]. The fine particles can penetrate deep into the alveoli, entering the blood stream through pulmonary capillaries, and their relatively large and reactive surface area allows them to carry other harmful substances. Once in the body, PM_2.5_ can have multiple effects including systemic and local lung inflammation, oxidative stress and DNA damage, and swelling and increased permeability of mucous membranes in the airways [10].

In a prior ecological time series analysis, we found an increase in injury-related deaths during the mine fire in geographical sites most affected by the mine fire, and that all-cause and cardiac-related deaths increased in the following 6 months [11]. However, a limitation of this analysis was that mine fire smoke exposure had to be determined by residential area, which could not account for important individual-level confounding factors such as pre-fire health status, tobacco use, and socioeconomic deprivation.

While the existing evidence has largely settled the question of whether PM_2.5_ increases risk of mortality, it typically focuses on urban background PM_2.5_ [6, 7]. We know of no studies of the long-term effects of discrete but extreme exposures such as those experienced during the Hazelwood coal mine fire. In this article, we build on our previous work by analysing a linkage between survey data collected from participants in the Hazelwood Health Study Adult Cohort [12] and estimates of individual-level exposure, as well as the Australian National Death Index (NDI) [13] to identify deaths within the cohort. We addressed the following research question: did smoke exposure from the mine fire increase long-term risk of all-cause, cardiac, cancer, or respiratory-related mortality?

## Materials and methods

### Study cohort

**Figure 1:**
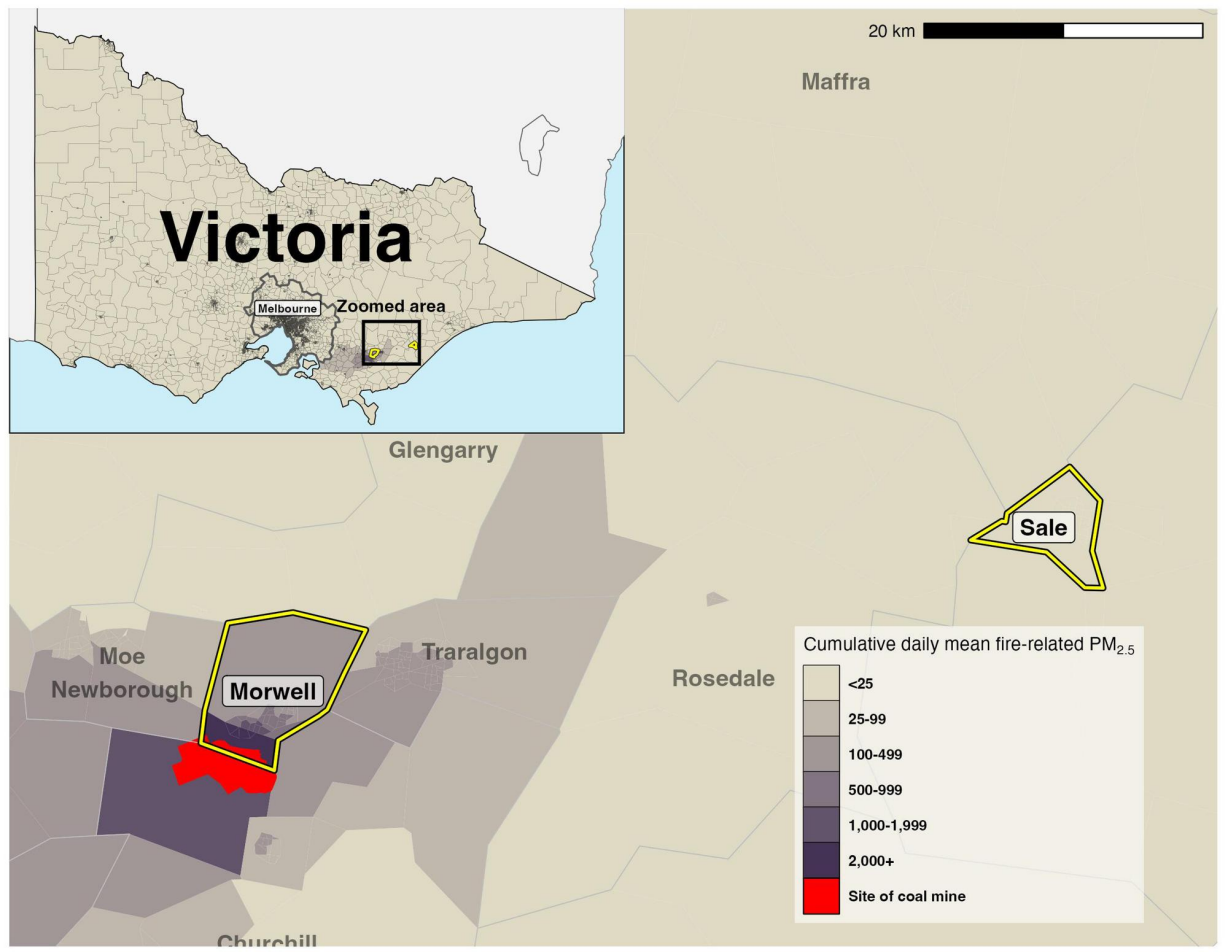
Map of mine fire area with distributions of cumulative fine particulate matter ≤2.5 µm (PM_2.5_), highlighting the exposure site of Morwell and control site, Sale.

The Hazelwood Health Study Adult Cohort was recruited between May 2016 and February 2017. Prospective participants were identified using the electoral roll maintained by the Victorian Electoral Commission to sample people residing in two towns in eastern Victoria (electoral registration is compulsory for Australian citizens aged ≥18 years). The first town was Morwell, adjacent to the mine fire and most severely affected by smoke exposure (exposed group). A control group was recruited among those who were living in 16 statistical areas at level one (SA1s) of Sale, a town around 65 kms away. The 16 areas of Sale were selected for similarity to Morwell in terms of median age, household size, socioeconomic factors, and population stability. In Fig. 1, we present a map of the mine fire area with distributions of cumulative PM_2.5_, highlighting the study sites.

Data from air pollution modelling showed that Sale had minimal smoke exposure during the mine fire [3]. Residents who had been living in the pre-defined locations during the mine fire were sent postal invitations to participate in the Adult Survey, along with information leaflets about the study. In addition to completing the Adult Survey, participants were invited to provide written informed consent to link their data with NDI. More information about the Adult Cohort is available in our cohort profile [12].

### Exposure

The Hazelwood coal mine ignited on 9 February 2014; while the Victorian Country Fire Authority declared it ‘under control’ 10 days later, it was only deemed ‘safe’ on 25 March 2014 [14]. We generated individual-level fire-related PM_2.5_ exposure measures using a combination of modelled air quality data and time-location diaries, covering 9 February–31 March 2014. Due to a lack of air quality monitoring during the mine fire in Morwell and its surrounds, particularly in the earliest days when smoke levels were at their most intense [1], it was necessary to generate estimates of PM_2.5_ distributions. The Commonwealth Scientific and Industrial Research Organization (CSIRO) [3] applied chemical transport and meteorological models to estimate temporal and spatial distributions; this factored in the type of coal found in Victorian mines and that it was an open-cut mine. In the final model, geospatial resolution was as precise as 100 × 100 m in areas near the mine, decreasing in precision with distance. The estimated PM_2.5_ data correlated reasonably well with the actual air quality data from the nearby air quality stations, and were most accurate for stations closest to the site of the fire, which recorded the highest exposures.

The modelled PM_2.5_ estimates were then combined with time-location diaries completed by cohort members during the Adult Survey, which documented their whereabouts during the mine fire period in 12-h intervals, to calculate the daily mean over the mine fire period. More information about this process can be found in previous publications [12, 15].

### Outcome

Mortality was measured in survival days by linking the cohort survey to the National Death Index (NDI) [13] up to June 2023. The NDI included information on cause of death using ICD-10 codes; those used in this study included cardiac (ICD-10: I00–I99, G45, G46), cancer (C00–C97, D45–D47) and respiratory-related mortality (J00–J99). However, cause of death information in the NDI lags substantially behind death notifications and was only available up to December 2021. To identify potential cause-specific mortality where cause of death was not available, a predictive model was developed using a nested cross-validated (outer loop: five-fold and inner loop: 10-fold) XGBoost algorithm [16]. More information is available in the Supplementary Materials.

### Risk and moderating factors

The following were included in analyses as both risk or moderating factors (see ‘Statistical Analysis’), all measured at the time of the survey: age, sex, educational attainment (‘secondary up to year 10’, ‘secondary years 11–12’, ‘certificate/diploma/tertiary degree’), study site (Morwell/‘exposure’, Sale/‘control’), smoker status (‘current’, ‘former with at least 100 lifetime cigarettes’, or ‘never’), cigarette pack-years [17], and self-reported pre-fire comorbidities (angina, myocardial infarction, heart failure, stroke, chronic obstructive pulmonary disease [COPD], cancer, or diabetes). To control for socioeconomic differences between participants, we also included the Index of Relative Socioeconomic Advantage and Disadvantage (IRSAD) score for each participant’s Statistical Area Level 1 (SA1) based on 2016 census data [18].

### Statistical analysis

Effects of fire-related PM_2.5_ exposure on all-cause mortality were evaluated with Cox proportional-hazards models [19], while differences in cause-specific mortality were evaluated with a Fine-Gray competing risk survival model, which accounted for censoring due to deaths from other causes [20]. The Fine-Gray model assumes that all those who have not experienced the event of interest, or have experienced a competing risk, are still in the risk set when estimating hazard rates, even if the competing risk is death from another cause. Hazard ratios are interpreted as the weighted average of time-varying hazard ratios [21].

Survival was measured from the end of the exposure window (31 March 2014) until the end of follow-up linkage with NDI data (27 June 2023). Analyses adjusted for risk factors that were added to an initial crude model in the following order: demographics (age, sex), SES (educational attainment, IRSAD score), study site, tobacco use, and comorbidities. To determine whether any risk factors influenced the relationship between exposure and outcome, we performed a series of moderator analyses, incorporating interactions between exposure and the moderator, using covariates from the fully-adjusted model. To enhance interpretability, continuous moderators were standardized into *z*-scores and the daily mean fire-related PM_2.5_ across the mine fire period was expressed in units of, and centred at, 10 µg/m³.

Missing data were addressed with random forest multiple imputations [22], each of which was separately analysed and pooled according to Rubin’s rules [23]. The number of imputations was equivalent to the proportion of cases with missing data (10 imputed datasets). All analyses were conducted in R [24] with RStudio [25]. Cleaning and analytical code are available on our public repository [26].

## Results

### Description

Of 4056 Adult Survey cohort members, 2223 from Morwell (72%) and 649 (68%) from Sale consented to the linkage of their survey responses with NDI data. Participants from both towns had similar age and sex distributions and similar levels of cigarette smoking exposure (pack-years) at the time of the survey. However, Sale participants had slightly higher levels of education and were more socioeconomically advantaged than those from Morwell (Table 1). As expected, Morwell residents had much higher estimated PM_2.5_ exposure from the mine fire, with a median of 11 μg/m^3^ [7 μg/m^3^, 19 μg/m^3^], while the median in Sale was 0 [0 μg/m^3^,0 μg/m^3^]. In total, during the 6–7 years of follow-up, 245 deaths occurred among residents of Morwell and 73 among residents of Sale (totalling 11% of the combined cohort). The proportion of cardiac deaths was higher in Morwell (3.4%–2.3%), but not significantly so (*P *= .200); other causes of death are listed in Supplementary Table S1, in the supplementary material.

**Table 1:** Descriptive statistics by study site.^a^^,^^b^

	Morwell	Sale	
	*n *= 2223	*n *= 649	*P*-value
Daily mean exposure to fire-related PM_2.5_ (µg/m³)	11 (7–19)	0 (0–0)	
All-cause mortality	245 (11%)	73 (11%)	.887
Cause of death^c^			
Cardiac	75 (3.4%)	15 (2.3%)	.200
Actual	55 (2.5%)	12 (1.8%)	
Predicted	20 (0.9%)	3 (0.5%)	
Cancer	87 (3.9%)	16 (2.5%)	.092
Actual	59 (2.7%)	14 (%22)	
Predicted	28 (1.3%)	2 (0.3%)	
Respiratory	20 (0.9%)	8 (1.2%)	.495
Actual	17 (0.8%)	8 (1.2%)	
Predicted	3 (0.1%)	0 (0.0%)	
Survived	1978 (89%)	576 (89%)	
Age at survey	60 [IQR: 48–70]	60 [IQR: 45–71]	.871
Male	1025 (46%)	277 (43%)	.128
Educational attainment			**<.001**
Secondary to year 10	708 (32%)	148 (23%)	
Secondary to year 12	439 (20%)	107 (16%)	
Certificate/diploma/tertiary degree	1062 (48%)	391 (61%)	
Missing	14	3	
IRSAD^d^ scores	833 [IQR: 782–889]	898 [IQR: 844–952]	**<.001**
Missing	59	0	
Smoking status			**.009**
Non-smoker	1028 (47%)	341 (53%)	
Former smoker	802 (36%)	215 (33%)	
Current smoker	379 (17%)	87 (14%)	
Missing	14	6	
Smoking status			**.009**
Non-smoker	1028 (47%)	341 (53%)	
Former smoker	802 (36%)	215 (33%)	
Current smoker	379 (17%)	87 (14%)	
Missing	14	6	
Cigarette pack-years (current/former smokers)	15 [IQR: 5–30]	15 [IQR: 5–30]	.711
Missing	25	4	
Any pre-fire comorbidity	659 (30%)	177 (27%)	.259

a Continuous data presented by median and inter-quartile range (compared with Kruskal–Wallis tests); dichotomous/categorical data presented by number and percent (compared with Fisher’s exact tests).

b *P*-value is based on comparison of combined predicted and actual causes of death.

c Cause of death predicted for *n *= 93 who had died in the most recent 18 months for which death notification was available but cause was not—predictions were made separately for each cause-specific outcome, with overlap in predicted cause for two individuals (one person predicted as both CVD and cancer, another as both cancer and respiratory).

d Index of Relative Socioeconomic Advantage and Disadvantage.

Abbreviations: PM_2.5_ = fine particulate matter ≤2.5 μm; μg/m^3^ = micrograms per cubic metre.

### Main results

**Figure 2:**
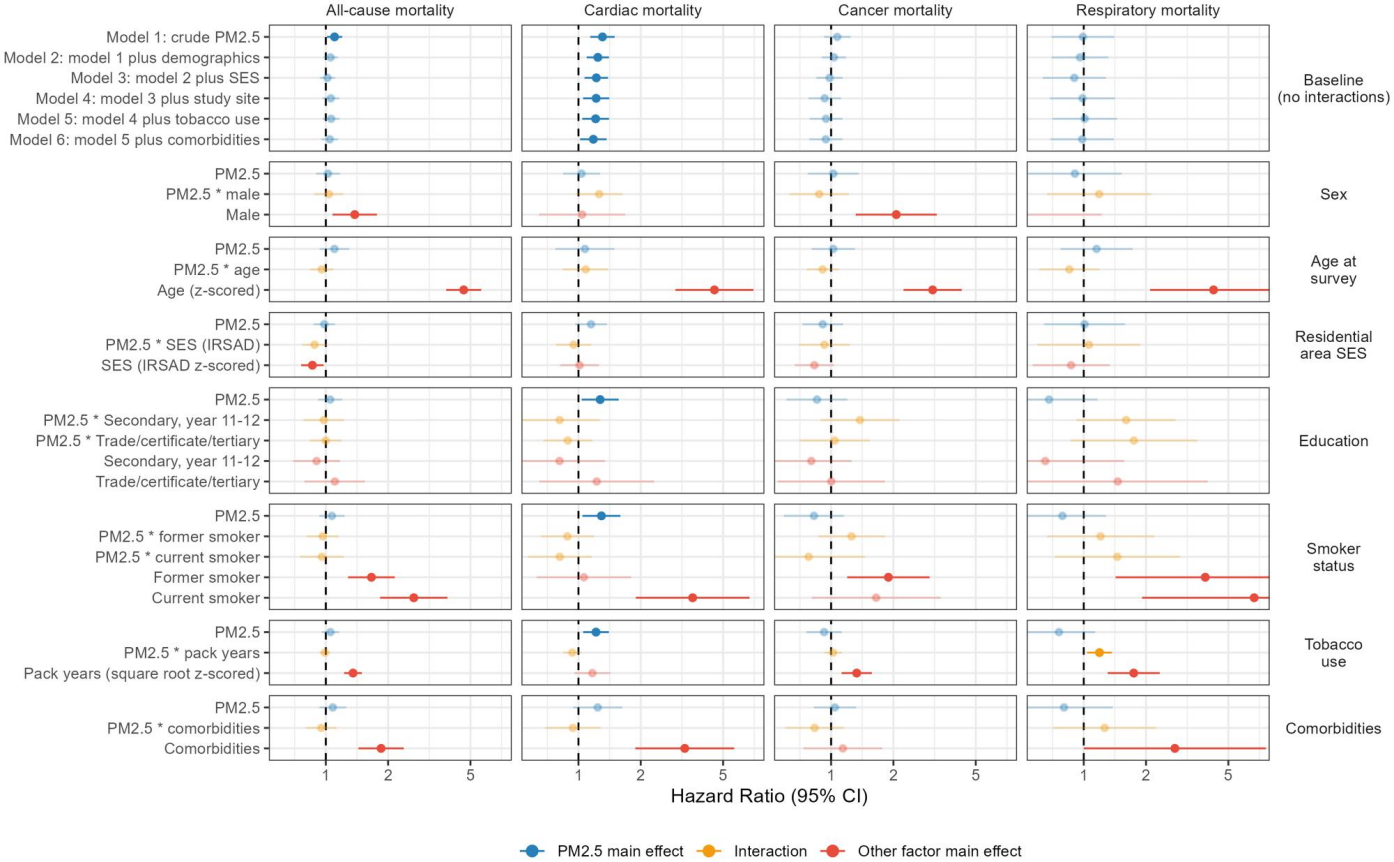
Forest plot of fine particulate matter ≤2.5 µm (PM_2.5_) (per 10 µg/m^3^ increase) effects on mortality, from crude to adjusted models, and moderated effects in fully-adjusted models.

Figure 2 and Supplementary Table S2 in the supplementary material summarize the results of the Cox proportional hazards (all-cause mortality) and competing risks (cardiac, cancer, and respiratory mortality) survival models, providing estimated hazard ratios (HRs) and 95% confidence intervals for each model. PM_2.5_ exposure had no detectable effect on all-cause mortality except in the crude (unadjusted) model. Likewise, there were no detectable effects on cancer mortality.

However, there was a detectable effect of PM_2.5_ exposure and risk of cardiac mortality. The association attenuated with each additional adjustment in baseline models (no interactions), though confidence intervals remained above 1, with the smallest point estimate being an HR of 1.18 (95% CI 1.02–1.37) per 10 µg/m^3^ of fire-related PM_2.5_ (fully-adjusted model 6). There was no detectable effect of PM_2.5_ on respiratory mortality, although there was an interaction between cigarette pack-years (square root transformed) and PM_2.5_ (HR: 1.19; 95% CI: 1.04–1.37). This suggested that exposure to PM_2.5_ from the mine fire exacerbated the effect of tobacco use on the risk of respiratory-related death. There were no detectable interactions between PM_2.5_ and smoker status (current, former, never) on respiratory mortality, although the point estimates for each were positive.

## Discussion

We found a crude association between mine fire-related PM_2.5_ exposure and all-cause mortality in the 9 years following the mine fire, but this attenuated to null after adjustment for confounders. There were no detectable associations of mine fire smoke with cancer mortality, a finding which aligned with the results of our previous analysis of anonymous Victorian Cancer Registry data [27]. However, neither of these findings should be taken as conclusively ruling out long-term mortality effects, which may have been masked by limited statistical power.

While there were no detectable effects of fire-related PM_2.5_ on respiratory mortality, we found evidence of a synergistic effect with tobacco use. Similar interactions between tobacco use and background PM_2.5_ have previously been observed for all-cause [28], cardiovascular [29], and lung cancer mortality [30], though to the best of our knowledge, this had not been previously examined for non-malignant respiratory mortality. It remains plausible that PM_2.5_ from this discrete, but extreme, fire event had a main effect on respiratory mortality, especially given the evidence that other sources of PM_2.5_ increase long-term respiratory morbidity [31], but our ability to detect a main effect was limited by the small number of respiratory deaths.

We found a robust association between PM_2.5_ exposure and risk of cardiac mortality in the 9 years following the mine fire. This aligns with the current body of evidence on the long-term effects of landscape fires on cardiovascular mortality [31]. Notably, we found no evidence that this association was moderated by demographics, socioeconomic factors, smoking, or comorbidities. This contrasted with previous findings about the combined effects of PM_2.5_ and smoking on cardiovascular death [29], although this, too, may be due to limited statistical power.

To date, there has been limited research about the mortality caused by pollution from coal mine fires [32]. Liu *et al.* undertook a systematic review of the health effects of different types of acute exposure to outdoor air pollution, namely that caused by forest fires, wildfires, and peat fires [33]. Including studies published between 1986 and 2014, the authors identified 13 studies of mortality after wildfire smoke exposure, nine of which showed increased rates of all-cause mortality. Unfortunately, Liu *et al.* identified no studies of cardiac mortality specifically, but identified 14 studies which considered cardiac morbidity following acute exposure to wildfire smoke, assessed by presentations or admissions with cardiac arrest or symptoms of a cardiovascular disease after the exposure occurred [33]. However, there is evidence that PM_2.5_ exposure from landscape or wildfires is deadlier than background PM_2.5_ that typically originates from sources like industrial processes and traffic [8]. Interestingly, the review by Liu *et al.* reported some geographic variation in associations. Five out of six studies from the USA found wildfire smoke exposure was associated with increased hospital admissions for cardiovascular diseases, while none of the seven studies from Australia and Canada found an effect on cardiovascular morbidity [33]. Only one other study in Porto, Portugal, found cardiovascular hospital admissions increased for over three months of summer forest fires in 2005 [34]. Liu *et al.* noted that rates of cardiovascular disease are much higher in the USA than the other countries from which studies originated (Australia and Canada) [33], which may have increased vulnerability to wildfire smoke effects.

What is most notable about the two effects we observed in this study—fire-related PM_2.5_ increasing risk of cardiac deaths and exacerbating the effects of smoking on respiratory deaths—is that both emerged in a long-term follow-up that began with recruitment into the cohort 2–3 years *after* exposure. This raises other issues (see discussion of immortal time bias below), but the main implication of our findings is that discrete, but intense, fire events like the Hazelwood coal mine fire could have long-lasting health effects, even increasing the risk of death, well after the exposure. While the literature on long-term effects of smoke exposure is limited, it is not without precedent. Former smokers have elevated risk of all-cause mortality as well as death from cancer, cardiovascular disease, and lower respiratory infections [35], indicating the mortality risks from respiratory toxins can persist well after the exposure has ended.

Although our findings are concordant with the growing body of literature, they were still unexpected, given that the participants in this cohort study could only be recruited 2–3 years after the coal mine fire. We previously demonstrated there was an excess of deaths from cardiovascular disease associated with PM_2.5_ exposure within 6 months of the mine fire [11]. This likely introduced immortal time bias into the cohort, where early deaths attributable to the mine fire depleted the exposed community of its most susceptible members before they could join the cohort [36]. For this reason, we suggest that the excess cardiac mortality as well as respiratory mortality among smokers reported here are likely under-estimated.

This study has some strengths: the analyses were based on an established cohort for whom we had collected considerable information about individual risk factors, supplemented with diaries about their movements during the mine fire period. This enabled individual-level estimation of their PM_2.5_ exposure. Furthermore, the data about mortality have been extracted up to 9 years after the mine fire from administrative databases, providing objective and comprehensive data that are known to be accurate and take account of deaths occurring outside the State of Victoria.

However, these findings must be considered alongside some limitations. Although the total follow-up period has been 9 years following the mine fire, the number of deaths, particularly cause-specific deaths, was relatively small, limiting the statistical power of the current analyses. For this reason, further follow-up is still needed to know for sure whether deaths from all-causes have increased, and to more precisely estimate the additional cardiac deaths caused by exposure to the coal mine fire. Also, as noted above, the cohort was established following an increase in deaths during and shortly after the mine fire, introducing immortal time bias; the implication is our reported effects are likely to be underestimates.

In summary, our findings suggest that extreme smoke events like the Hazelwood coal mine fire have long-lasting harms. In the wake of such disasters, those with pre-existing cardiovascular problems should be considered vulnerable and given special care for protection during smoke events and monitoring afterwards.

## Supplementary Material

dyag071_Supplementary_Data

## Data Availability

Hazelwood Health Study data are confidential and cannot be publicly shared but are available per reasonable request. Analytical code has been archived on a public repository.
