# Supplementary material for: Mortality in relation to smoke exposure 9 years after the Hazelwood coal mine fire
Source: Int J Epidemiol. 2026 May 12;55(3):dyag071. doi: 10.1093/ije/dyag071 (PMC13166877; doi:10.1093/ije/dyag071)
Supplement: dyag071_Supplementary_Data [file dyag071_supplementary_data.pdf]

Mortality in relation to smoke exposure nine years after the Hazelwood coal mine fire: supplementary materials

Table of Contents

1 Supplementary tables 2

2 Fire-related $PM_{2.5}$ distribution 4

2.1 Fire-related $PM_{2.5}$ exposure distributions within the study cohort 5

3 Prediction model for unknown causes of death 6

3.1 Example: prediction of cardiac deaths 6

4 References 8

1 Supplementary tables

Table S1. Cause of death by study site

Cause of death	Morwell (*n* = 245)	Sale (*n* = 73)	Total (*n* = 318)
Cancer	59 (24.1%)	14 (19.2%)	73 (23.0%)
Cardiovascular disease	55 (22.4%)	12 (16.4%)	67 (21.1%)
Respiratory system	17 (6.9%)	8 (11.0%)	25 (7.9%)
External cause	7 (2.9%)	2 (2.7%)	9 (2.8%)
Nervous system	5 (2.0%)	4 (5.5%)	9 (2.8%)
Genitourinary system	8 (3.3%)	1 (1.4%)	9 (2.8%)
Diabetes	6 (2.4%)	2 (2.7%)	8 (2.5%)
Digestive system	4 (1.6%)	4 (5.5%)	8 (2.5%)
Other causes with less than 5 cases	10 (4.1%)	7 (9.6%)	17 (5.3%)
Missing	*74 (30.2%)*	*19 (26.0%)*	*93 (29.2%)*

Table S2. Associations between fire-related $PM_{2.5}$ exposure and mortality in the Hazelwood Health Study Adult Cohort

	All-cause mortality		Cardiac mortality		Cancer mortality		Respiratory mortality	
	HR (95% CI)	*P*-value	HR (95% CI)	*P*-value	HR (95% CI)	*P*-value	HR (95% CI)	*P*-value
**Baseline models (no interaction)**								
Model 1: crude fire-related $PM_{2.5}$ exposure	**1.10 (1.01-1.20)**	**0.024**	**1.31 (1.14-1.50)**	**<0.001**	1.07 (0.92-1.24)	0.365	0.99 (0.70-1.41)	0.961
Model 2: model 1 plus demographics	1.05 (0.97-1.14)	0.196	**1.24 (1.10-1.40)**	**0.001**	1.03 (0.90-1.18)	0.647	0.96 (0.70-1.31)	0.795
Model 3: model 2 plus SES	1.02 (0.94-1.11)	0.668	**1.22 (1.07-1.39)**	**0.003**	0.98 (0.85-1.14)	0.828	0.90 (0.63-1.28)	0.561
Model 4: model 3 plus study site	1.06 (0.96-1.16)	0.243	**1.22 (1.05-1.41)**	**0.008**	0.93 (0.78-1.11)	0.437	0.99 (0.69-1.42)	0.945
Model 5: model 4 plus tobacco use	1.06 (0.97-1.17)	0.207	**1.21 (1.04-1.40)**	**0.012**	0.95 (0.79-1.14)	0.558	1.01 (0.71-1.46)	0.942
Model 6: model 5 plus comorbidities	1.04 (0.95-1.15)	0.362	**1.18 (1.02-1.37)**	**0.027**	0.94 (0.79-1.14)	0.546	0.98 (0.69-1.40)	0.927
**Age model**								
Fire-related $PM_{2.5}$ exposure	1.10 (0.94-1.30)	0.246	1.07 (0.78-1.49)	0.666	1.03 (0.81-1.30)	0.835	1.16 (0.77-1.73)	0.482
$PM_{2.5}$ * age at Survey (z-score)	0.95 (0.84-1.09)	0.482	1.08 (0.84-1.39)	0.534	0.91 (0.76-1.09)	0.303	0.85 (0.61-1.19)	0.348
Age at Survey (z-score)	4.65 (3.83-5.65)	**<0.001**	4.59 (2.98-7.07)	**<0.001**	3.10 (2.24-4.28)	**<0.001**	4.21 (2.08-8.52)	**<0.001**
**Sex model**								
Fire-related $PM_{2.5}$ exposure	1.02 (0.90-1.17)	0.716	1.04 (0.84-1.27)	0.737	1.03 (0.77-1.36)	0.856	0.91 (0.54-1.53)	0.712
$PM_{2.5}$ * male	1.03 (0.88-1.21)	0.688	1.26 (0.97-1.63)	0.085	0.88 (0.63-1.22)	0.433	1.19 (0.67-2.12)	0.558
Male	**1.38 (1.08-1.76)**	**0.010**	1.05 (0.65-1.70)	0.842	**2.05 (1.30-3.22)**	**0.002**	0.51 (0.21-1.22)	0.128
**Comorbidities model**								
Fire-related $PM_{2.5}$ exposure	1.09 (0.94-1.26)	0.263	1.24 (0.94-1.63)	0.125	1.04 (0.82-1.32)	0.722	0.82 (0.48-1.40)	0.468
$PM_{2.5}$ * comorbidities	0.94 (0.79-1.12)	0.496	0.94 (0.69-1.28)	0.696	0.83 (0.60-1.16)	0.275	1.23 (0.70-2.17)	0.477
Any comorbidities	**1.87 (1.46-2.41)**	**<0.001**	**3.26 (1.88-5.65)**	**<0.001**	1.13 (0.73-1.74)	0.597	**2.92 (1.06-8.07)**	**0.038**
**Index of Relative Socioeconomic Advantage and Disadvantage 2016**								
Fire-related $PM_{2.5}$ exposure	0.98 (0.87-1.11)	0.780	1.15 (0.96-1.37)	0.123	0.92 (0.73-1.15)	0.438	1.01 (0.65-1.58)	0.963
$PM_{2.5}$ * IRSAD score (z-score)	0.88 (0.77-1.01)	0.074	0.94 (0.77-1.15)	0.565	0.93 (0.70-1.24)	0.621	1.06 (0.60-1.88)	0.845
IRSAD score (z-score)	**0.86 (0.76-0.98)**	**0.020**	1.02 (0.82-1.27)	0.881	0.83 (0.67-1.03)	0.088	0.87 (0.57-1.34)	0.535
**Educational attainment (ref: secondary up to year 10)**								
Fire-related $PM_{2.5}$ exposure	1.05 (0.92-1.20)	0.458	**1.28 (1.04-1.57)**	**0.020**	0.86 (0.61-1.20)	0.378	0.68 (0.40-1.17)	0.161
$PM_{2.5}$ * Secondary year 11-12	0.98 (0.78-1.22)	0.847	0.81 (0.52-1.26)	0.352	0.81 (0.52-1.26)	0.352	1.38 (0.89-2.13)	0.151
$PM_{2.5}$ * certificate/diploma/tertiary	0.99 (0.83-1.18)	0.931	0.88 (0.67-1.16)	0.367	0.88 (0.67-1.16)	0.367	1.03 (0.70-1.53)	0.876
Secondary year 11-12	0.91 (0.70-1.18)	0.475	0.81 (0.49-1.35)	0.423	0.82 (0.52-1.27)	0.369	0.66 (0.27-1.57)	0.344
Certificate/diploma/tertiary	1.11 (0.79-1.55)	0.539	1.22 (0.65-2.32)	0.536	1.01 (0.56-1.84)	0.970	1.48 (0.54-4.05)	0.442
**Smoker status at survey**								
Fire-related $PM_{2.5}$ exposure	1.07 (0.93-1.23)	0.359	**1.28 (1.04-1.59)**	**0.020**	0.83 (0.59-1.15)	0.265	0.79 (0.48-1.28)	0.340
$PM_{2.5}$ * former smoker	0.97 (0.81-1.16)	0.727	0.90 (0.67-1.20)	0.474	1.25 (0.86-1.82)	0.235	1.20 (0.66-2.18)	0.542
$PM_{2.5}$ * current smoker	0.96 (0.75-1.22)	0.711	0.81 (0.57-1.16)	0.245	0.78 (0.42-1.46)	0.435	1.45 (0.72-2.91)	0.296
Former smoker	**1.65 (1.28-2.14)**	**<0.001**	1.04 (0.61-1.75)	0.894	**1.90 (1.20-3.00)**	**0.006**	**3.87 (1.43-10.50)**	**0.008**
Current smoker	**2.68 (1.85-3.90)**	**<0.001**	**3.63 (1.92-6.88)**	**<0.001**	1.66 (0.81-3.40)	0.167	**6.66 (1.91-23.20)**	**0.003**
**Cigarette pack-years at survey**								
Fire-related $PM_{2.5}$ exposure	1.05 (0.95-1.16)	0.305	**1.22 (1.05-1.40)**	**0.007**	0.93 (0.76-1.13)	0.444	0.76 (0.51-1.14)	0.185
$PM_{2.5}$ * pack-years (square root)	0.99 (0.93-1.05)	0.750	0.93 (0.84-1.03)	0.160	1.02 (0.93-1.13)	0.633	**1.19 (1.04-1.37)**	**0.013**
Pack-years (square-root)	**1.35 (1.22-1.49)**	**<0.001**	1.16 (0.94-1.42)	0.165	**1.34 (1.13-1.58)**	**0.001**	**1.74 (1.30-2.33)**	**<0.001**

2 Fire-related $PM_{2.5}$ distribution

Using fire-related $PM_{2.5}$ estimates from Luhar et al. 2020[1], we plotted distributions of cumulative exposures both in the main manuscript (Figure 1) and below (Figure S1) by Statistical Area at Level 1 (SA1). SA1 is a geographic unit created by the Australian Bureau of Statistics that is based around communities of around 400 (range: 200-800) residents[2].

Along with Figure 1 in the main manuscript, the rug plot along the bottom of Figure S1 clearly shows that Morwell (yellow) is the most exposed site in Victoria, while the control site of Sale (red) is in the middle of the rest of Victoria (blue). Note that the x-axis has been log-transformed for interpretability, though this also de-emphasises how extreme the exposures in Morwell were compared to the rest of the Victoria.

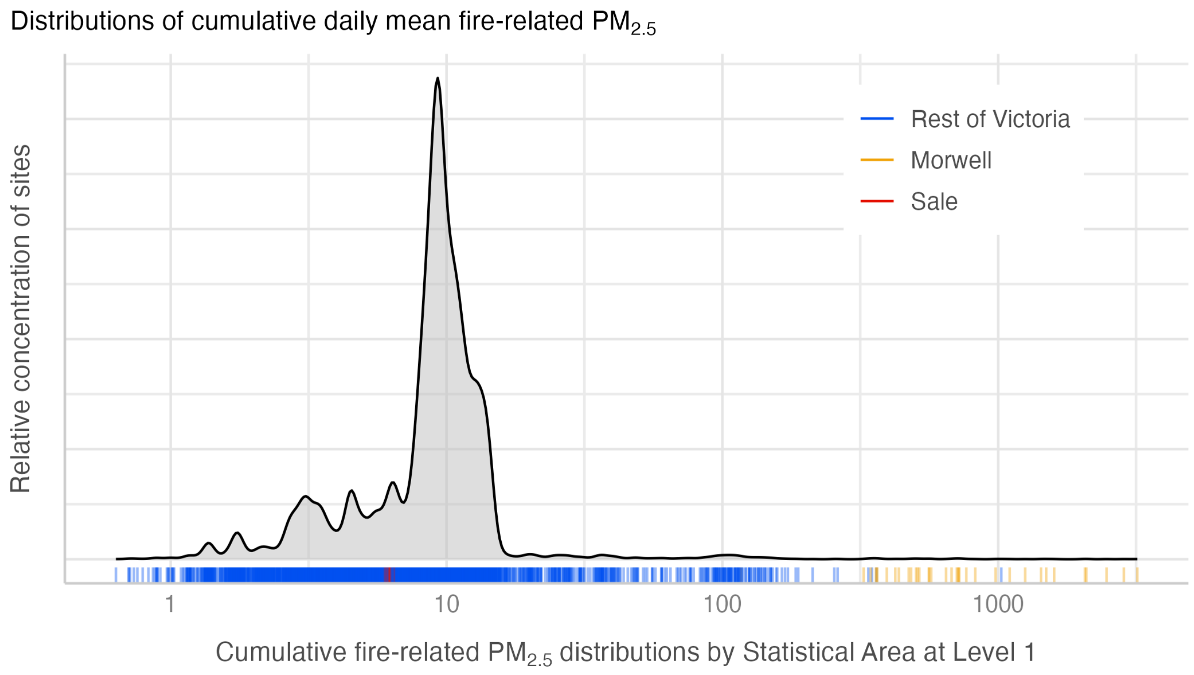

Figure S1. Distributions of fire-related fine particulate matter ≤2.5μm ($PM_{2.5}$) exposure across Victoria by Statistical Area at Level 1[2]

2.1 Fire-related $PM_{2.5}$ exposure distributions within the study cohort

While we refer to Morwell as our "exposure" site, there are considerable variations in daily fire-related $PM_{2.5}$ exposure between individuals. This was the product of our efforts to create individual-level exposure measures that were sensitive to daily movements during the mine fire period (see *2.2 Exposure* in the manuscript, Luhar et al. (2020)[1], Johnson et al. (2020)[3], and Ikin et al. (2021)[4] for more detail). The full range of fire-related $PM_{2.5}$ exposures in Morwell was 0.004-56μg/m^3 [IQR: 7-19μg/m^3]; in Sale, the same figures were 0-4μg/m^3 [IQR: 0-0μg/m^3].

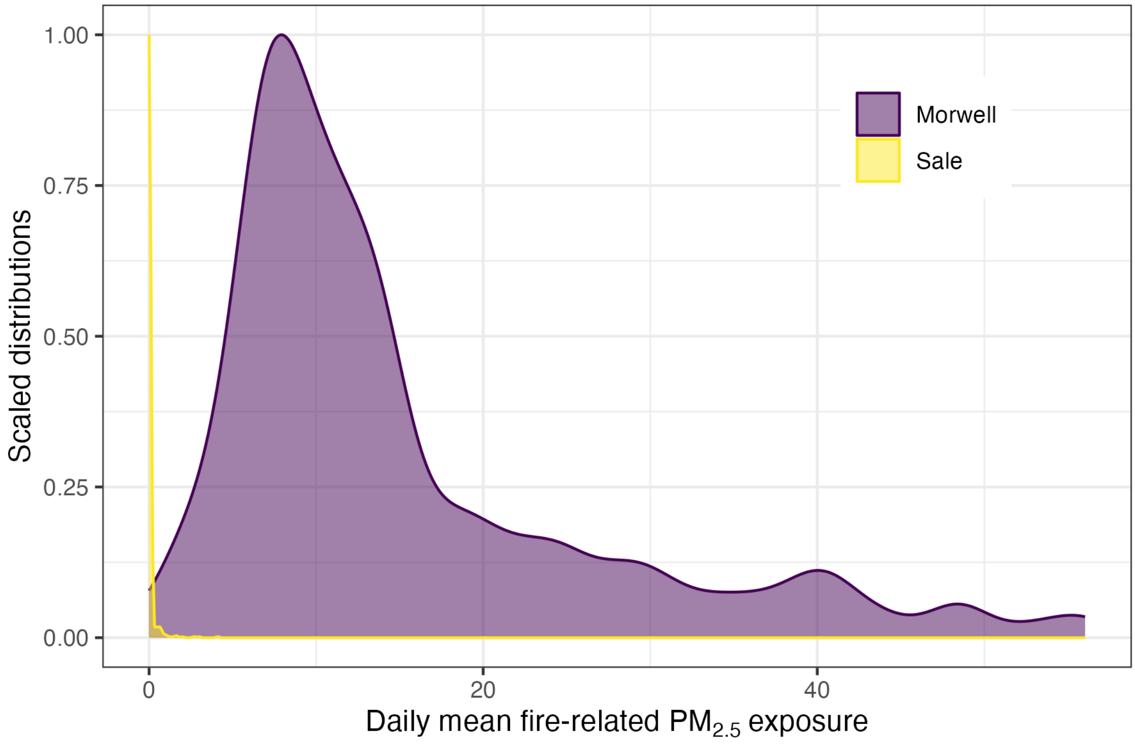

Figure S2. Distributions of daily mean fire-related fine particulate matter ≤2.5μm ($PM_{2.5}$) exposure within the Hazelwood Health Study Adult Survey cohort, grouped by exposure (Morwell) and control (Sale) sites

3 Prediction model for unknown causes of death

Survey data were linked to National Death Index (NDI)[5] deaths up to June 2023. The underlying cause of death was available from the NDI up to, and including, deaths until December 2021. We predicted likely cardiac (ICD-10: I00-I99, G45, G46), cancer (C00-C97, D45-D47), and respiratory-related mortality (J00-J99) among those lacking a cause using the methods described below.

This prediction model took advantage of the data-linkage study design and obtained potential predictors from the Adult Survey[4] (e.g., demographics, smoking, alcohol consumption, socioeconomic status, psychological distress, and self-reported doctor-diagnosed conditions). The model included predictors extracted from three linked healthcare records: the Victorian Admitted Episodes Dataset (VAED) for hospital admissions[6], the Victorian Emergency Minimum Dataset for emergency presentations[7], and Ambulance Victoria electronic patient care records for ambulance attendances up to 30 June 2022[8]. These predictors included the total number of healthcare services utilised, as well as service utilisation for specific condition groups (classified by ICD-10 chapters and corresponding ambulance diagnostic groups), within the 5 years preceding the mortality event. Due to the high dimensionality of the data, we first ran the prediction model to identify the top 20 predictors and re-trained it with selected predictors to improve model accuracy.

3.1 Example: prediction of cardiac deaths

The cardiac mortality prediction model with a total of 225 observations, including 67 known cardiac deaths as training data, had a cross-validation Area Under the Curve of 0.76. With a probability cut-off of 0.45, the algorithm had a sensitivity of 0.98 and specificity of 0.80 on the training data. The model predicted an additional 23 cardiac deaths among 93 new mortality records that did not specify a cause of death. The variable importance from the final prediction model is provided in Figure S3, which indicates mine fire exposure is one of the key predictors differentiating between cardiac and non-cardiac deaths.

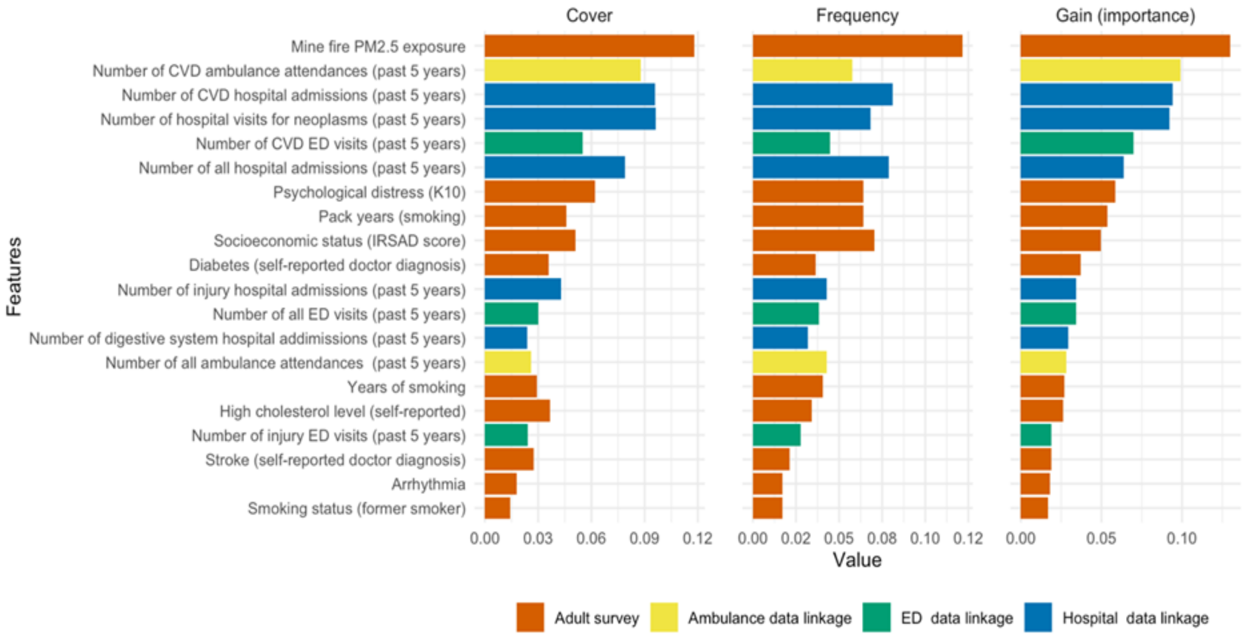

Figure S3. Variable importance plot of the top 20 factors in the prediction model for cardiac mortality among all mortality data.
Figure glossary – *Cover*: relative quantity of observations that a feature splits; *Frequency*: the number of times a feature is used to split data across all trees in the model; *Gain*: the improvement in accuracy (or reduction in loss) brought by a feature when it is used to split the data in the decision tree; *ED*: hospital emergency department; *CVD*: cardiovascular disease.

4 References

1. Luhar AK, Emmerson KM, Reisen F, Williamson GJ, Cope ME. Modelling smoke distribution in the vicinity of a large and prolonged fire from an open-cut coal mine. *Atmos Environ*. 2020 May;**229**:117471.

2. Australian Bureau of Statistics. Statistical Area Level 1 | Australian Bureau of Statistics [Internet]. 2021 [cited 2025 Sept 1]. Available from: https://www.abs.gov.au/statistics/standards/australian-statistical-geography-standard-asgs-edition-3/jul2021-jun2026/main-structure-and-greater-capital-city-statistical-areas/statistical-area-level-1

3. Johnson AL, Gao CX, Dennekamp M, et al. Coal-mine fire-related fine particulate matter and medical-service utilization in Australia: a time-series analysis from the Hazelwood Health Study. *Int J Epidemiol*. 2020 Feb 1;**49**(1):80–93.

4. Ikin J, Carroll MTC, Walker J, et al. Cohort profile: The Hazelwood Health Study Adult Cohort. *Int J Epidemiol*. 2021 Jan 23;**49**(6):1777–1778.

5. National Death Index (NDI) [Internet]. *Australian Institute of Health and Welfare* 2023 [cited 2023 Dec 13]. Available from: https://www.aihw.gov.au/about-our-data/our-data-collections/national-death-index

6. Department of Health (Victoria). Victorian Admitted Episodes Dataset [Internet]. State Government of Victoria, Australia; 2023 [cited 2023 May 17]. Available from: https://www.health.vic.gov.au/data-reporting/victorian-admitted-episodes-dataset

7. Department of Health (Victoria). Victorian Emergency Minimum Dataset (VEMD) [Internet]. State Government of Victoria, Australia; 2023 [cited 2023 May 17]. Available from: https://www.health.vic.gov.au/data-reporting/victorian-emergency-minimum-dataset-vemd

8. Cox S, Martin R, Somaia P, Smith K. The development of a data-matching algorithm to define the ‘case patient’. *Aust Health Review*. 2013;**37**(1):54.
